# Differential Trypanocidal Activity of Novel Macrolide Antibiotics; Correlation to Genetic Lineage

**DOI:** 10.1371/journal.pone.0040901

**Published:** 2012-07-31

**Authors:** Carolina Aquilino, Maria Luisa Gonzalez Rubio, Elena Maria Seco, Leticia Escudero, Laura Corvo, Manuel Soto, Manuel Fresno, Francisco Malpartida, Pedro Bonay

**Affiliations:** 1 Centro de Biología Molecular “Severo Ochoa”, Universidad Autónoma de Madrid, Campus de Cantoblanco, Madrid, Spain; 2 Centro Nacional de Biotecnología, Campus de Cantoblanco, Madrid, Spain; Federal University of São Paulo, Brazil

## Abstract

Here we report the systematic study of the anti-trypanocidal activity of some new products derived from *S. diastatus* on 14 different *T. cruzi* strains spanning the six genetic lineages of *T. cruzi*. As the traditional growth inhibition curves giving similar IC_50_ showed great differences on antibiotic and lineage tested, we decided to preserve the wealth of information derived from each inhibition curve and used an algorithm related to potency of the drugs, combined in a matrix data set used to generate a cluster tree. The cluster thus generated based just on drug susceptibility data closely resembles the phylogenies of the lineages derived from genetic data and provides a novel approach to correlate genetic data with phenotypes related to pathogenesis of Chagas disease. Furthermore we provide clues on the drugs mechanism of action.

## Introduction

Chagas disease or American Trypanosomiasis is a chronic illness caused by the Kinetoplastid protozoan *Trypanosoma cruzi*. At present it represents the third largest parasitic disease burden in the world and the largest in the western hemisphere. It is endemic in Central and South America with over 10 million people currently affected, with 40–100 million (15–20%of the whole Latin American population) at risk of acquiring the disease, killing around 15000 people annually [1], [2].

In the past decades, mainly because of increased population movements, the number of diagnosed cases has increased also in non-endemic countries in the Region of the Americas (Canada and United States of America), and in Europe (principally in Spain, Belgium, France, Italy, Switzerland and United Kingdom of Great Britain and Northern Ireland) [3]–[5] and the Western Pacific regions (Australia and Japan). This increase presents additional risks of transmission of the parasite through blood transfusion, congenital infection and organ transplantation [6].

The complex pathogenesis of the disease starts with a low mortality initial acute phase with generally mild and unspecific symptoms in which Th1 lymphocytes, IFN-gamma and macrophages are in charge of controlling parasite replication [7]–[9].This leads to a long lasting chronic condition in which the cellular immune responses fight to unsuccessfully limit parasite proliferation and elimination, resulting in a sustained inflammatory response that triggers the development of one or more of the diverse and geographically divergent symptomatic clinic forms of the disease that emerges in 20–40% of patients such as chronic chagasic cardiomyophaty (CCC), digestive problems like megaesophagous and megacolon and neuropathies [7], [10]–[14], which may appear decades after the initial infection.

It remains unexplained why different patients develop the cardiac, digestive, cardio-digestive or indeterminate clinical forms. One possible clue is a significant geographical variation in the prevalence and severity of the different forms of the disease. It is believed that this geographical heterogeneity is caused primarily by genetic variation of *T. cruzi*, since it does not present a clear correlation with any pattern of ethnic or human genetic or environmental variables [15].

The parasite role on the acute phase pathology is unquestionable; however there have been controversy over the years on its influence on its participation in the chronic phase pathogenesis [16]–[19], but the consensus growing over the least years is that the persistence of parasites (clonal or mixed population), coupled with an unbalanced immune response leads in susceptible individuals combined to infecting strain(s) virulence, to a sustained inflammatory responses that underlies the lesions of chronic Chagas disease. This implies that eradication of *T. cruzi* parasites in the host tissue is an ineludible condition to arrest the evolution of the disease.

Efficacy of current chemotherapy of Chagas disease is controversial as the two drugs in use, Nifurtimox (NFX; Lampit®) and Benznidazol (BZN, Rochagan®, Radanil®) have severe side effects [20]–[23], and are at the best moderately effective in the chronic stages of infection, besides requiring long courses of treatment without specific pediatric formulations [24], [25] and there is a great deal of different activity towards the different strains belonging to the six genetic lineages of *T. cruzi* [26], without any attempts to establish correlations [27]–[30].

Over the last three decades, a great effort has been made to reduce the vectorial and transfusional transmission of Chagas disease in some endemic areas through the interruption of reservoir to human and human to human propagation [31], however, there is consensus that this neglected illness cannot be totally eradicated with the current measure and intervention tools. Thus, it is urgent to continue the development of new therapeutic tools and to carry out surveys of new anti-trypanocidal drugs on a wide array of strains as a complementary way to evaluate biochemical distinctive traits that can be correlated to the clinical forms of the disease.

Our group reported recently the antifungal activity of a new macrolide antibiotic [33] CE-108 from *Streptomyces diastaticus* 108 with some activity against *T. cruzi* [34]. Here we report the systematic study of the anti-trypanocidal activity of some new products derived from *S. diastatus* on 14 different T. cruzi strains representing the six genetic lineages of *T. cruzi* [26] and provide clues on the action mechanism. Furthermore, preserving the wealth of information derived from each inhibition curve, we used an algorithm related to potency of the drugs on epimastigotes, combined in a matrix data set used to generate a cluster tree that closely resembles the phylogenies of the lineages derived from genetic data.

## Materials and Methods

### Cells and Parasites

Vero (green monkey kidney cells, ATCC CCL-81) and LLC-MK2 (Rhesus kidney cells, ATCC CCL-7) cell lines were both obtained from ATCC and grown in 24 wells plates in RPMI medium supplemented with 7.5% FCS, humidified 5% CO_2_.

The different strains of *Trypanosoma cruzi* used are indicated in Table 1, and were obtained through ChagasEPInet Consortia (http://www.ki.se/chagasepinet/), and grown as epimastigotes in RPMI +10% FCS at 28°C, by passages every five days. Metacyclic trypomastigotes forms of selected strains were obtained from the supernatant of infected Vero cells and isolated by differential centrifugation.

**Table 1:** Relation of strains used on this study, with adscription to genetic lineage, geographical origin and host from which was isolated.

Strain	Lineage	Origin	Host/Vector
Silvio/X10 c1	TcI	Belem, Brazil	*Homo sapiens*
C8 c1	TcI	La Paz, Bolivia	*Triatoma infestans*
Dm28c	TcI	Carabobo, Venezuela	*Didelphis marsupialis*
Esmeraldo c3	TcII	Bahia, Brazil	*Homo sapiens*
Y	TcII	Sao Paulo, Brazil	*Homo sapiens*
Tu18 c2	TcII	Tupiza, Bolivia	*Triatoma infestans*
Cm17	TcIII	Carimaga, Colombia	*Dasyprocta fugilinosa*
M6241 c6	TcIII	Para, Brazil	*Homo sapiens*
10R26	TcIV	Santa Cruz, Bolivia	*Aotus sp.*
Bug2148 c11	TcV	Rio Grande do Sul, Brazil	*Triatoma infestans*
Sc43 c1	TcV	Santa Cruz, Bolivia	*Triatoma infestans*
Tula c2	TcVI	Tulahuen, Chile	*Homo sapiens*
VFRA c1	TcVI	Francia, Chile	*Triatoma infestans*
CL-Brener	TcVI	Rio Grande do Sul, Brazil	*Triatoma infestans*

All indicated strains were obtained through the ChagasEPINet Consortia (http://www.ki.se/chagasepinet/).

### Infection Assays

Vero or LLC-MK2 cells growing on glass coverslips at 25% confluency in 24 well plates were infected with tripomastigotes at an infection index of 10 during for 4 hours. The cells were washed three times to remove unattached parasites and kept at 37°C with or without drugs for the indicated times and concentrations.

At indicated times; cells were washed twice with PBS, fixed with Bouińs fixative solution and stained in Giemsa solution. The intracellular amastigotes were quantified counting randomly at least 300 cells.

### Drugs

The drugs used (AB-400 (AB), Pimaricin (PIM), CE-108B, CE-108D, CE-108E) were obtained and purified as described before [33]–[36]. Briefly, when needed, the polyenes were weighted, dissolved in dimethyl sulphoxide (DMSO) and diluted with medium up to the required concentration immediately prior to each experiment. The final DMSO concentration in the culture never exceeded 0.2%.

Benznidazole (Rochagan) was provided by M. Miles at the London School of Hygiene and Tropical Medicine, London, UK.

### Toxicity and Viability Assays

For anti-trypanocidal assays on epimastigotes, the parasites (50000 cells, mid-log) were grown in 96 wells plates in a volume of 200 microliters to which the drugs were added at the indicated final concentrations and the growth followed during seven days by measuring Optical Density at 600 nm. Control wells containing media and vehicle of the drugs (DMSO) were included. Three replicate plates were used for each drug. The drug concentration that inhibits 50% growth was estimated by plotting the parasite numbers at different days as a percentage over dug concentration.

Metacyclic trypomastigote forms (1×10^6^ cells/ml) were maintained at 37°C in RPMI medium supplemented with 10% FBS and then incubated for 24 h with different concentrations (1–100 µM) of the drugs, final volume 100 µl in microtiter plates. The IC_50_ (concentration that lyses 50% of the parasites) was then evaluated by counting the cells in a Neubauer chamber. Each test was made in two experiments conducted in duplicate.

To evaluate the toxicity and viability effects of the drugs on mammalian cells the MTT assay (3-[4,5-dimethylthiazol-2-yl]-2,5-diphenyl tetrazolium bromide) was used as reported [37] on cells growing in 96 wells plates.

Once toxicity on cells was determined, treatment with the drugs was performed by two protocols: (1) pretreatment: incubate cells with drugs during the four hour infection period and keep the drugs in media after washing the unattached parasites, and (2) add the drugs 24 hours post infection and keep them for the duration of the experiment.

### Electron Microscopy

For scanning electron microscopy, the parasites drug treated and control, were adhered to poly-L-lysine-coated coverslips, fixed with 2.5% glutaraldehyde in 0.1 M Na-cacodylate buffer (pH 7.2) at room temperature for 40 minutes and post-fixed with a solution of 1% OsO_4_, 0.8% potassium ferricyanide and 2.5 mM CaCl_2_ in the same buffer for 20 min. The cells were dehydrated in an ascending acetone series and dried by the critical point method with CO_2_. The samples were mounted in aluminium stubs, coated with a 20 nm thick gold layer and examined in a Hitachi S-3000N scanning electron microscope (SEM).

### Flow Cytometry

Epimastigotes (5×10^6^ cells/ml) were treated at the times and doses indicated at 28°C. Thereafter the cells were incubated for 15 min with 10 µg/ml tetramethyrhodamine methylesther (TMRM, Invitrogen) and kept on ice until analysis. Data acquisition and analysis were done on a FACSCalibur flow cytometer (Becton-Dickinson, CA, USA) and Cell Quest software. Ten thousand events were acquired. Alterations in the fluorescence for TMRM were quantified using an index of variation (IV) obtained by the equation (M*_T_*-M*_C_*)/M*_C_* were M*_T_* is the median of fluorescence for treated parasites and M*_C_* that of control cells (Menna-Barreto, 2005). A negative value for IV corresponds to depolarization of the mitochondrial membrane. In some experiments we used rhodamine 123 (Rh123) as fluorescent probe obtaining the same results as with Rh123.

For analysis of viability and exposure of PS, the epimastigotes, after treatment with the drugs were stained with 10 µg/ml of propidium iodide (PI) and with 1 mg/ml annexin V-FITC, respectively, for 30 minutes. Data acquisition was done as described before.

### Clustering

The inhibition produced by each antibiotic on a given strain was measured by using an algorithm which is described [38] as:


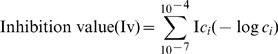


Where Ci represents each one of the micromolar antibiotic concentrations tested (0.1, 0.3, 1, 3, 10, 30, and 100) and *I*c_i_ the percentage of growth inhibition at the C_i_ concentration when compared with untreated control. The application of this algorithm yields dimensionless values for each pairwise strain-antibiotic. The pairwise values obtained were used to generate a cluster tree to show relationships between strains, Ward´s method, single linkage and complete linkage hierarchical cluster analysis methods were used to group strains, and a French PCA was run in Statistica version 8 (www.statsoft.com) to determine the similarities between the strains. To establish if a correlation exists between genetic distances (obtained from the literature [39], [40] and the Euclidean distances obtained in this report, a Mantel test [41] was used.

**Table 2:** Trypanocidal activity of antibiotics and Benznidazol (BZN) on epimastigote form.

Strain	PIM	CE108	CE108B	CE108D	CE108E	AB400	BZN
Silvio/X10 c11	0.8	3.5	2.1	4.2	3.9	3.2	31.8
C8	1.8	3.8	3.8	6.3	3.7	2.5	25.7
Dm28c	1.1	2.9	4	5.9	4.1	3.7	22.2
Esmeraldo c13	0.9	3.9	2.9	5.1	5.2	nd	30.5
Y	0.8	3.7	3.1	4.9	5.5	2.5	12.4
Tu18	1.2	4.1	3.2	5.4	4.8	2.7	15.1
Cm17	0.8	2.1	0.9	1.5	1.8	1.2	10.4
M6241 cl6	1.1	2.4	0.8	1.8	2	nd	12.1
10R26	10.5	8.1	12	7.8	9	10.2	22.4
Bug2148 c11	21	18	25	31	27	17	28.1
Sc43 c11	40	22	30	45	38	21	21.4
Tula	1.5	3.5	2.9	2.5	3.8	2.8	16.2
VFRA	0.9	2.8	3.5	5.2	6.1	4.3	11.2
CL-Brener	0.9	3.2	4.1	4.2	5.9	2.4	15.8

## Results

### Effect on Epimastigotes

**Figure 1:**
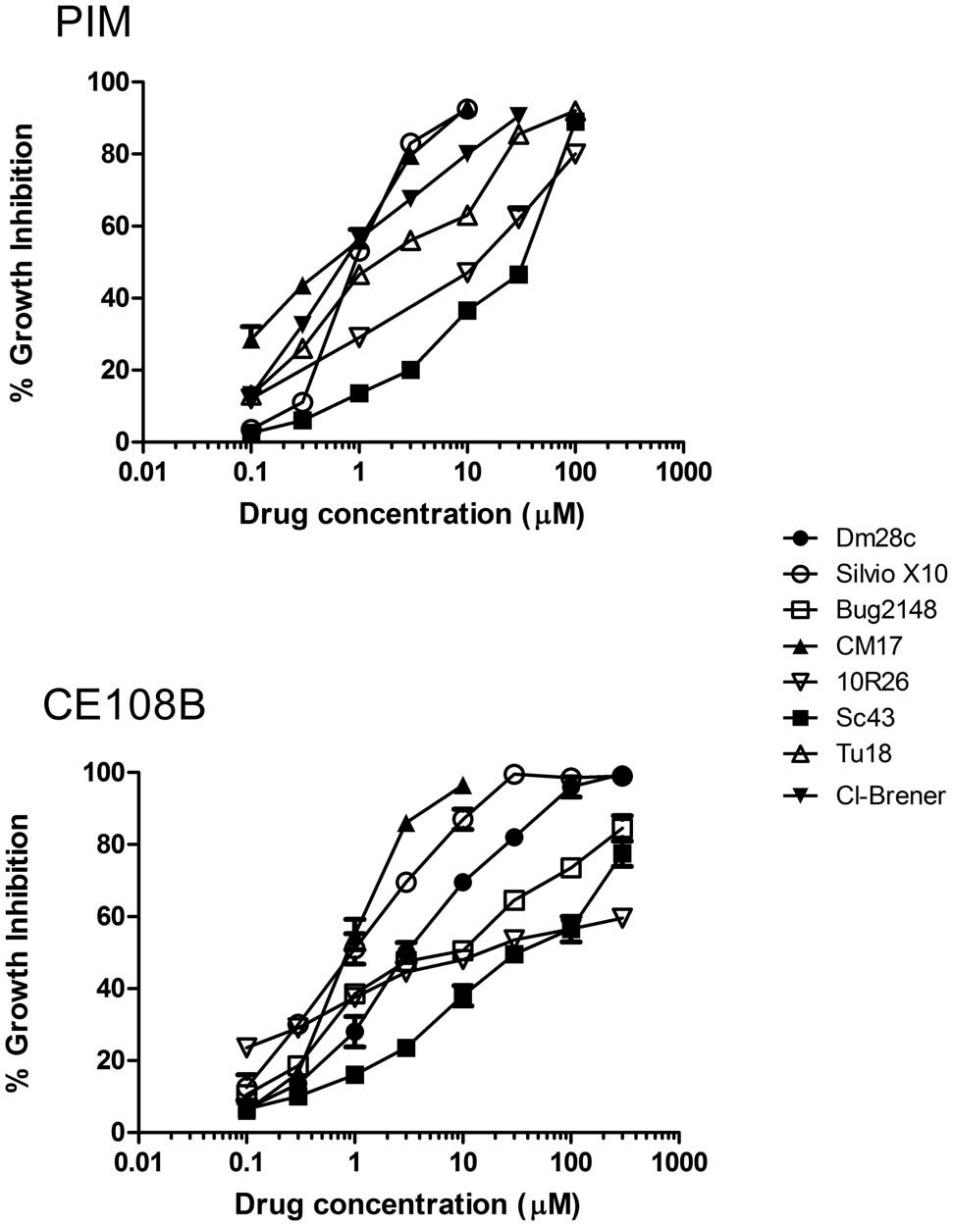
Representative dose-viability curves of PIM and CE108B on different DTUs after 72 hours incubation. Epimastigotes of indicated DTUs were incubated with the drug for 72 hours in the microtiter plate assay as described in Materials and Methods. It shows the growth inhibition compared to control cultures without any drug (shown media of three experiments in triplicate).

On Fig. 1 are shown representative dose dependent growth inhibition curves for some DTUs at 48 hours. All the strains indicated on Table 1 were susceptible to the antibiotics tested but differed remarkably on the shape of the dose dependence, indicating different susceptibilities thresholds or differential accumulative effects. Routinely, one out of two measures was done by counting cells also to corroborate correlation between OD and cell number. The Table 2 reports the IC_50_ for all strains against all antibiotics (and benznidazol as a control drug) tested at 48 hours.

### Effect on Metacyclic Trypomastigotes

We decided to test the activity of some selected antibiotics (PIM, CE108B, CE108E) on metacyclic trypomastigotes viability to validate any antitrypanocidal effect and to verify that any correlation data obtained from epimastigotes would remain valid to trypomastigotes. The data are summarized in Table 3 indicate that the antibiotics tested exhibit antitrypanocidal effects on that life form of the parasite. It is possible to establish a correlation between the effects seen against non-infective epimastigotes and those seen on metacyclic tripomastigotes, thus strains from DTUs IV and V (Sc43 and 10R26) were the less susceptible to the tested antibiotics, in contrast to DTUs I, II and VI that exhibit a greater susceptibility to the drugs. In any case, it is significative that trypomastigotes in all cases exhibited a higher susceptibility than the epimastigotes counterpart.

**Table 3:** Activity on metacyclic tripomastigotes.

Strain	PIM	CE108	CE108B	CE108D	CE108E	AB400	BZN
Silvio/X10	0.5	nd	0.8	nd	0.4	nd	12.4
Y	0.5	nd	1.8	nd	2.1	nd	8.1
CM17	0.8	nd	2.2	nd	0.5	nd	11.4
10R26	4	nd	3.5	nd	3.1	nd	16.2
Sc43 c11	14	nd	2.8	nd	7.2	nd	13.2
CL-Brener	0.5	nd	0.9	nd	0.3	nd	7.8

The table shows the effective concentration causing 50% growth inhibition at 72 hours compared to untreated control culture (IC_50_) in micromolar.

**Figure 2:**
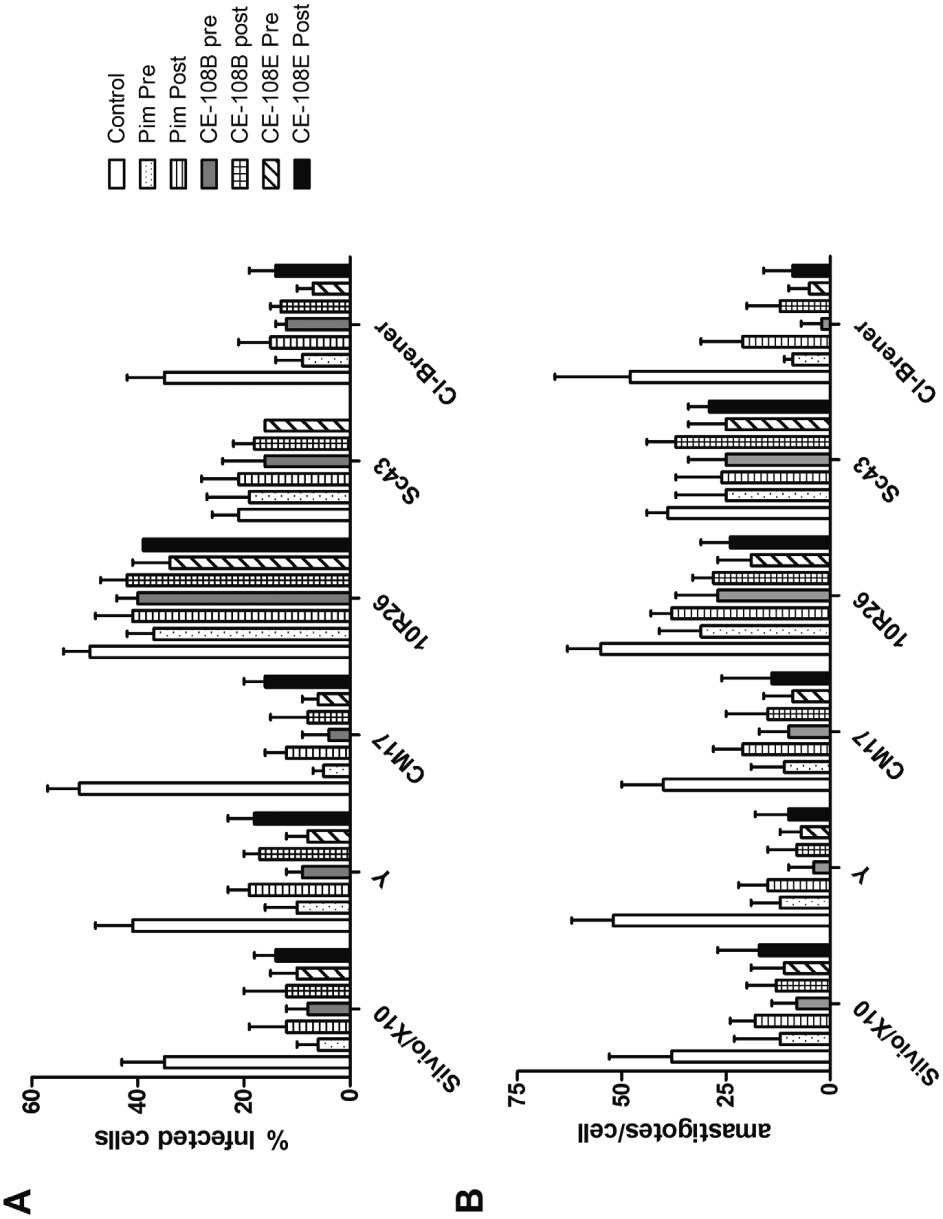
Effect of antibiotics on *in vitro* infection and intracellular amastigotes. LLC-MK2 cells were infected with bloodstream trypomastigotes of the indicated DTUs as detailed in Materials and Methods. Two different protocols were used, PRE: the drugs were added during the four hours infection period and kept during the proliferation of amastigotes intracellularly. In the second protocol (POST), the drugs were added to the culture medium 24 h after the infection. After four days, the cultures were processed as described and not less than 300 cells were counted by two independent observers. A.- Shows the percentage of infected cells. B.- Shows the number of amastigotes per infected cell.

### Cytotoxic Activity

Before analyzing the effect of the drugs on intracellular amastigotes we evaluated the toxicity on cultured mammalian host cell lines Vero and LLC-MK2. On both cell lines, the viability starts being compromised at concentrations above 100 µM with all the antibiotics tested at times above 48 hours of incubation. We observed that antibiotics CE108, CE108B and CE108D, always result less toxic to the cells when compared with AB and PIM. Due to sample limitations the toxic effect of CE108E was only tested at 10 µM, concentration at which exhibited a behavior similar to CE108B. At the concentrations effective on the epimastigotes growth and trypomastigotes viability (1–10 µM), the viability was never below 90% after 96 hours incubation, with the only exception of Vero cells treated with 10 µM PIM, that showed a viability of 80% after 96 h treatment.

**Figure 3:**
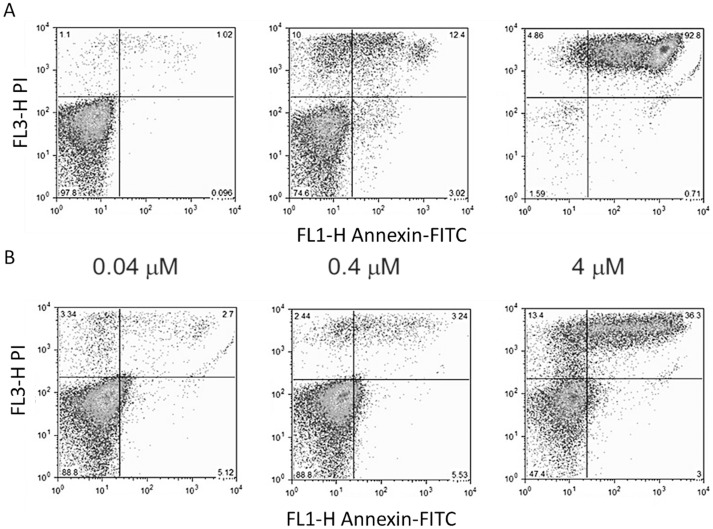
PS exposure and membrane permeability changes induced by the antibiotics. Epimastigotes of Silvio/X10 strain were treated with PIM (A) or CE108B (B) during 4 hours with the indicated drug concentrations. Representative dot-plots of parasites stained for PS exposure (Annexin V, X-axis) and membrane permeability (PI staining, Y-axis).

### Effect on Amastigotes

Once confirmed the low toxicity on host cells, the effect of CE108B, CE108E and Pim (the drugs with overall lower IC_50_) on metacyclic trypomastigotes and proliferating epimastigotes was evaluated on six strains of *T. cruzi*, Silvio/X10 (TcI), Y (TcII), CM17 (TcIII), 10R26 (TcIV), Sc43 (Tc V) and Cl-Brener (TcVI) infecting LLc-MK2 or Vero cells. It was evaluated following two protocols as indicated in Material and Methods. In one protocol, the drugs were added during the four hours infection period and kept during the proliferation of amastigotes intracellularly. In the second protocol, the drugs were added to the culture medium 24 h after the infection. The concentration used for testing efficacy was 5 µM in all cases. The cultures were evaluated 3 days after infection and the percentage of infected cells as well as the number of amastigotes per cell was quantified by counting not less than 300 cells. The results shown in Fig. 2 indicate that all three antibiotics tested are effective against the six strains evaluated. In all cases the pre-treatment, even for a short 4 hour period, resulted in a higher infection blocking efficacy, probably due to higher effective concentration affecting the ability of parasite to penetrate the cells and establish a productive infection. The efficacy ranking of the antibiotics on amastigotes development (measured as the difference in % of infected cells in treated vs. control cultures) could be correlated to the IC50 on proliferating epimastigotes, thus the DTUs IV and V showed to be less susceptible to the drugs. We were not able to find a significant difference on the number of amastigotes per cells between the two different protocols; however this was the case when the number of infected cells was considered, where the pretreatment resulted in a statistically significant lower number of infected cells.

**Figure 4:**
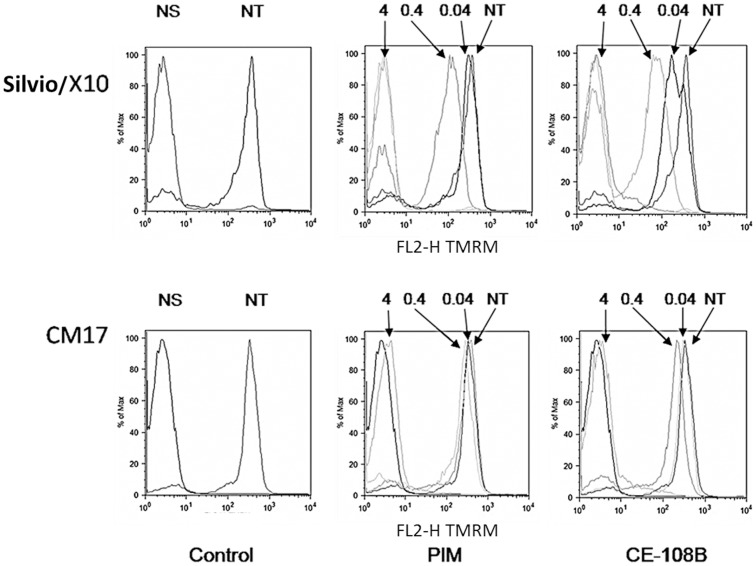
Antibiotic treatment caused loss of ΔΨm in epimastigotes. Representative histograms plots showing TMRM staining of Silvio/X10 and CM17 epimastigotes treated or not with PIM and CE108B during 4 hours at indicated concentrations in micromolar. NS: Not stained; NT: Not treated.

### Flow Cytometry

PS exposure in the outer leaflet of the plasma membrane is a common feature of eukaryotic cells undergoing death by apoptosis. By using annexin V-FITC and PI, PS exposure and membrane permeability of parasites exposed to the antibiotics were simultaneously accessed. Untreated parasites were annexin V and PI negative (not shown). The Fig. 3 shows a cytometry dose dependence profile of annexin-V and PI staining for Silvio/X10 strain at 4 h incubation with PIM (A) and CE108B (B). This is a representative profile common to all the strains and antibiotics tested, in which PIM is showing effect at lower concentrations, at least to what concerns to increase in permeability, and continue with exposure of PS.

In addition, alterations on the mitochondrial membrane potential of epimastigotes treated with different antibiotic concentrations for 4 hours were studied. Fig. 4 shows a representative experiment showing a drastic reduction of the median value of fluorescence of TMRM upon increasing antibiotic concentration indicating loss of mitochondrial potential of the epimastigotes. Table 4 presents a summary of *IV* values for the strains used for the two antibiotics tested in this analysis. It is possible to discriminate two behaviors regarding the threshold of effect, in some strains there is a gradual dose dependent effect on the membrane potential and on others there is sudden change from almost no effect to total dissipation of membrane potential when moving from 0.4 to 4 µM of antibiotic. The same behavior was noticed when the incubation time was increased to 24 hours (not shown).

**Table 4:** Summary of antibiotic treatment effect on the mitochondrial membrane potential.

Strain	Conc (µM)	*IV*	
		PIM	CE108B
Silvio/X10 c11	0.04	−0.28	−0.56
	0.4	−0.73	−0.82
	4	−0.99	−0.99
Y	0.04	−0.04	−0.06
	0.4	−0.09	−0.15
	4	−0.95	−0.98
Cm17	0.04	−0.02	−0.05
	0.4	−0.06	−0.45
	4	−0.88	−0.91
10R26	0.04	−0.04	−0.10
	0.4	−0.18	−0.21
	4	−0.35	−0.38
Sc43 c11	0.04	−0.02	−0.08
	0.4	−0.08	−0.12
	4	−0.19	−0.23
CL-Brener	0.04	−0.09	−0.1
	0.4	−0.32	−0.41
	4	−0.87	−0.91

IV: Index of variation as defined in Materials and Methods.

### Ultrastructural Analysis

**Figure 5:**
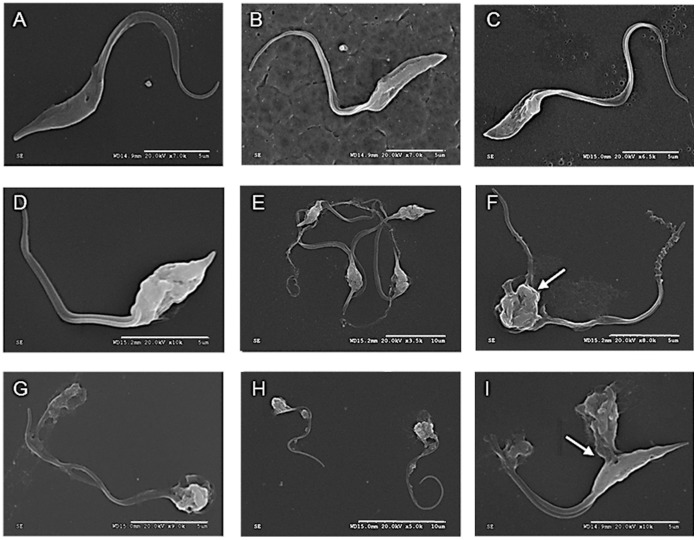
Ultrastructural studies of antibiotics effects. A. CM17 untreated control; B. Silvio/X10 untreated control; C. CM17 PIM 0.4 µM for 4 hours; D and E. Silvio/X10 PIM 0.4 µM for 4 hours; F. CM17 PIM 4 µM for 4 hours; G. Silvio/X10 CE108B 4 µM for 4 hours; H. CM17 PIM 0.4 µM for 24 h; I. Silvio/X10 PIM 0.4 µM for 24 hours; Bars in white represent 10 µm in F and I, and 5 µm in all others figures.

The parasites from strain CM17 and Silvio/X10 were treated with different dose for different times with Silvio/X10 and CE108B and analyzed by SEM. The Fig. 5a and b, presents the control parasites showing the typical elongated body, smooth surface with terminal flagellum. Treatment with PIM for 4 hours at 0.4 µM induce some ruffling and blebbing of the membrane as seen in Fig. 5c, d and e. Increasing the concentration at 4 µM, or the time at 0.4 µM to 24 h results in dramatic structural alterations as shown in Fig. 5f, h and i, in which it can be seen the parasite with large holes on the membrane from which loss of internal content is evident (arrow in panel i), body retraction and detachment of flagella. We did not identify differences in the kinetics of appearance of ultrastructural alterations among the strains examined. There are no significant differences in the morphological alterations upon CE108B (Fig. 5g).

**Figure 6:**
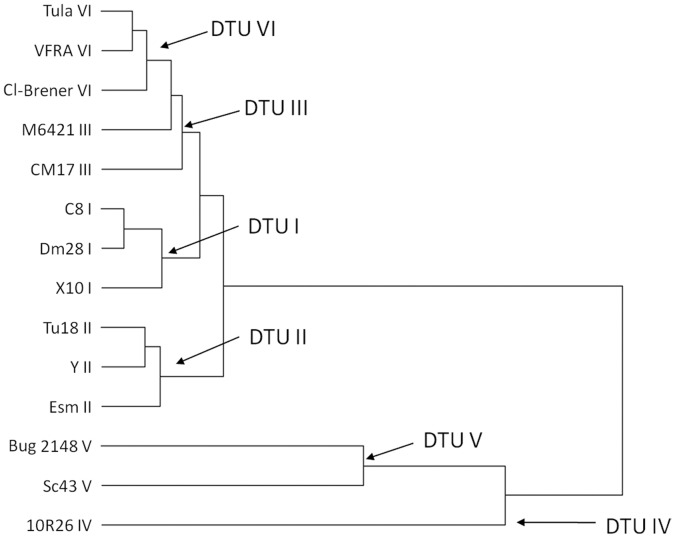
Cluster tree build from accumulated drug sensitivity as described in Materials and Methods.

### Functional Correlation

In order to gain some insight on a comparative functional analysis of the different lineages and take profit of the great deal of information stored on the growth inhibitory data (shape of curve, range of inhibition) we used the algorithm (see Material and Methods) that transforms each inhibition curve into a dimensionless value that represents *T. cruzi* strain sensitivity. The figure 6 shows the cluster obtained by using the Ward´s method analysis, comparing the sensitivity to different antibiotics (PIM, CE108, CE108B, CE108D and CE108E). The antibiotic AB400 was not included in the analysis as we do not have the full dose-response curves for all strains (for some strains we have just 4 concentrations instead of the seven-eight with the rest). BZN was excluded as the presumed action mechanism is different to the antibiotics described here, and no cross-resistance has been found in the literature (65). The results of such analysis reveal a correlation between the phylogenetic data based on sequences derived from miniexon, 16 S rRNA and population genetics, and the functional data reported here on antibiotic sensitivity. The figure shows that the strains pertaining to DTU V and IV (only strain 10R26) are clearly separated and distinct from the DTU I-III and VI. At the same time, DTU I and II are relatively well defined from each other as independent groups. The relationship between DTUs VI and III remains less clear, however the correlation between other genetic markers [39], [42]–[45] and the Euclidean distances is still significant (data not shown). Similar clusters were obtained by single or complete linkage analysis. However, the cluster obtained in this report does not reveal the same topology as the ones obtained by analysis of genetic markers [39], [40], [42], [44], [45].

## Discussion

The limitations and drawbacks of the current chemotherapy for Chagas’ disease highlight the determination for the identification and evaluation of novel trypanocidal compounds [23], . Here we present data on novel macrolide antibiotics derived from *Streptomyces diastaticus* that show higher anti-trypanosomal activity *in vitro* than the standard used drug Benznidazol, with significant lower IC_50_. These antibiotics exhibit activity against all stages of development with low toxicity on host cells. The effect on amastigotes was evaluated under two protocols, pretreatment with the antibiotics during the binding/attachment/infection period (pre), and treatment 24 h after initial infection (post). In both cases we observed a decrease of the percent of infection and of the number of intracellular amastigotes, but did not observe an alteration on the differentiation to trypomastigotes in treated cultures. The difference between pre and post-treatment may be attributed to higher active concentration of active compound and its effects on viability of parasite before or during the infection event. It is important to note that treatment within the time span of those experiments caused no damage to the cell lines studied as judged by microscopy and later confirmed by the toxicity assays.

The limited available data on the susceptibility on the amastigotes (only studied six and not the whole set of 14 strains), and the fact that we have just studied one concentration instead of the full inhibition curve, imposes some limitations on the final analysis and make comparisons difficult, however we can establish some correlation with the epimastigotes susceptibility. The same occurs with the metacyclic tripomastigotes, thus strains from DTUs IV and V are the ones that showed lower susceptibility to the drugs no matter the developmental stage studied. It is clear that a systematic and more detailed study on clinically relevant forms has to be made before any extrapolation of medical significance.

Considering the ultrastructural alterations, membrane blebbing is indicative of apoptosis-like; and plasma membrane rupture is indicative of necrosis [47]. The characterization of programmed cell death or apoptosis-like pathways in protozoa is under discussion [48]–[50], even when some key components directly involved in the proteolytic machinery that mediates apoptotic cell death have been identified in such organisms [51]–[55] with not yet defined functional involvement [56]. We have observed both alterations suggesting interplay of different cell death pathways in the antibiotics effect on epimastigotes; however a more detailed study by transmission electron microscopy would need to be performed before assuming a conclusion. Mitochondria play important roles in both the life and death of cells, as not only involves respiration, but also in the death signaling pathways, induced by several stimuli [57]. The treatment with the antibiotics reported here provokes a drastic drop in fluorescence of both TMRM and Rh123 (not shown) suggesting interference with proton electrochemical potential gradient of the mitochondrial membrane at short times incubation leading most probably to organelle damage. The reduced retention of the fluorescent probes was not entirely due to plasma membrane permeabilization as the percentage of PI positive cells at some treatment conditions was not higher than 5% in cases in which there was a reduction on the IV value from control to −0.56, but cannot totally ruled out. However, in some of the images of SEM, it can be seen abnormal internal structures protruding; the largest subcellular structure in T. cruzi is the mitochondria. As we stated before, transmission electron microscopy analysis have to be performed in order to confirm damage to this organelle and others like Golgi apparatus or Endoplasmic Reticulum and disclose the trypanocidal action of those compounds.

The shape of the dose-effect curves were different fort each antibiotic and cell strain, so we wanted to take profit of that stored information. With that objective we implemented the algorithm already described [38] to cluster all the strains analyzed here based on the novel macrolide antibiotic susceptibility.

The results, showed an organization of the tested strains in groups that closely resembles the up to now six groups [26] obtained by genetic methods derived from sequences of diverse markers and named genetic lineages or more appropriately Discrete Typing Units (DTUs) [15], [58], [59].

The clonal nature of *Trypanosoma cruzi* postulated some years ago [60], [61] put forward that a correlation between the phylogenetic divergence of the clones and their biological properties may exist. Under this premise several reports have appeared exploring possible links between the biological diversity of *T. cruzi* and some biological properties like tissue tropism, virulence, infectivity, drug (benznidazol or nifurtimox) susceptibility or resistance, replication rates, metacyclogenesis rate [62], vector and geographical distribution and even membrane markers, like the trans-sialidase [63], gp90 [64] or Peanut agglutinin ligands [65]. Regarding drug susceptibility/resistance, is the biological property that most attention has received due to the therapeutic/clinical relevance. Several reports [66]–[68], sometimes conflicting [28], [66]–[68], using variable but normally non-reference or low number of strains, and more important, most of the work have been done before the now accepted classification on six lineages, just discriminating the two original genetic groups. It is now well established that former Tc II group is greatly heterogeneous comprising at least five diverse subgroups [15], [26], [45].

In contrast to previous studies, the data presented here builds on firm phylogenetic framework to explore functional diversity and the strains used have been genotyped and extensively characterized [58], including the normally poorly represented sylvatic DTUs TcIII and TcIV.

Similar clustering was obtained with either single linkage and complete linkage was used to group the strains (data not shown). It is clear from our result that indeed is possible to correlate a biological property, in this case the sensitivity to a drug (most probably associated to a genetic trait like lipid composition of the plasma membrane), quantified by the cumulative effect over a dose range, and not by a single parameter like the IC_50,_ with the genetic lineage. That way the whole information on the dose effect curve is taken in account and not only a single dose, in which case, threshold effects or cumulative effects are dismissed.

The results derived from this study, no matter the potential medical significance, cannot be extrapolated to other drugs as the responses to those would depend mainly on the action mechanism, compensatory metabolic changes and detoxification systems specific for each drug. In *Trypanosoma cruzi* has been demonstrated that there is no *in vivo* cross-resistance to nitroheterocyclic drugs (like BZN and nifurtimox) and imidazole derivatives (65) and under that premise, the currently used drug for Chagas’ disease, Benznidazol, was excluded from the joint analysis. The same reason would be valid in case any future analysis with BZN reveals a different cluster organization of the strains. Even when we were tempted to do a cluster analysis with our data set from BZN susceptibility data, they were limited enough to guarantee a significant result and will be done as soon as we get supply of BZN.

Our results are in partial agreement with a recent report [40] that has used a proteomic approach to identify DTU-specific proteins and dissimilarities to correlate phylogenetic character mapping in *T. cruzi*, as we also found a closer relationship between DTUs VI and III, than to the other DTUs between them. This is congruent with the phylogeny of *T. cruzi* established by other markers, however, the cluster obtained in this report does not reveal the same topology as those [39], [40], [42], [44], [45], in particular the closer distances between DTU I and II. But in any case there is a strong coincidence, supporting the notion that evolution of metabolic and biochemical properties are far from being independent of evolution of genetic characters. Results from our group based on a hierarchical analysis of the glycans family (terminal fucose and enzymes responsible for that) exposed on the surface of *T. cruzi* strains (Bonay et al, manuscript in preparation) supports that view.

Even when the main objective of this study was not to establish a phylogenetic mapping based on a biochemical/cell biology property as is the drug sensitivity. The results obtained in this report and the ones from Telleria et al. [40] provide the basis and a conceptual framework to continue the analysis of population structure of this complex organism and furthermore, to study biochemical traits associated to fundamental genetic traits like the membrane sterol composition of the different DTUs.

Our results strongly stimulate further studies on the efficacy testing of these novel antibiotics in a murine model of *T. cruzi* infection with different strains to correlate functional properties *in vivo* and also encourage carrying out similar studies on clinically relevant forms of the parasite by testing drugs currently in use or undergoing clinical trials.

## References

[1] Salvatella R (2007) Andean subregional Chagas disease area and the Andean initiative of Chagas disease. Mem Inst Oswaldo Cruz 102 Suppl 1: 39–40. 17906804 10.1590/s0074-02762007005000105

[2] WHO (2010) Chagas disease (American trypanosomiasis) fact sheet (revised in June 2010). Wkly Epidemiol Rec 85: 334–336. 20726172

[3] Bern C, Montgomery SP (2009) An estimate of the burden of Chagas disease in the United States. Clin Infect Dis 49: e52–54. 19640226 10.1086/605091

[4] Gascon J, Bern C, Pinazo MJ (2010) Chagas disease in Spain, the United States and other non-endemic countries. Acta Trop 115: 22–27. 19646412 10.1016/j.actatropica.2009.07.019

[5] Piron M, Verges M, Munoz J, Casamitjana N, Sanz S, et al (2008) Seroprevalence of Trypanosoma cruzi infection in at-risk blood donors in Catalonia (Spain). Transfusion 48: 1862–1868. 18522707 10.1111/j.1537-2995.2008.01789.x

[6] Schmunis GA, Yadon ZE (2010) Chagas disease: a Latin American health problem becoming a world health problem. Acta Trop 115: 14–21. 19932071 10.1016/j.actatropica.2009.11.003

[7] Coura JR, Borges-Pereira J (2010) Chagas disease: 100 years after its discovery. A systemic review. Acta Trop 115: 5–13. 20382097 10.1016/j.actatropica.2010.03.008

[8] Kayama H, Takeda K (2010) The innate immune response to Trypanosoma cruzi infection. Microbes Infect. 10.1016/j.micinf.2010.03.005 20348008

[9] Junqueira C, Caetano B, Bartholomeu DC, Melo MB, Ropert C, et al (2010) The endless race between Trypanosoma cruzi and host immunity: lessons for and beyond Chagas disease. Expert Rev Mol Med 12: e29. 20840799 10.1017/S1462399410001560

[10] The Lancet (2009) Chagas disease: the forgotten American neuroinfection. Lancet Neurol 8: 501. 19446265 10.1016/S1474-4422(09)70113-9

[11] Anonymous (2009) Chagas’ disease and its toll on the heart. Eur Heart J 30: 2063–2065. 19723697 10.1093/eurheartj/ehp277

[12] Biolo A, Ribeiro AL, Clausell N (2010) Chagas cardiomyopathy–where do we stand after a hundred years? Prog Cardiovasc Dis 52: 300–316. 20109600 10.1016/j.pcad.2009.11.008

[13] Matsuda NM, Miller SM, Evora PR (2009) The chronic gastrointestinal manifestations of Chagas disease. Clinics (Sao Paulo) 64: 1219–1224. 20037711 10.1590/S1807-59322009001200013 PMC2797592

[14] Rassi Jr A, Rassi A, Marin-Neto JA (2009) Chagas heart disease: pathophysiologic mechanisms, prognostic factors and risk stratification. Mem Inst Oswaldo Cruz 104 Suppl 1: 152–158. 19753470 10.1590/s0074-02762009000900021

[15] Macedo AM, Oliveira RP, Pena SD (2002) Chagas disease: role of parasite genetic variation in pathogenesis. Expert Rev Mol Med 4: 1–16. 10.1017/S1462399402004118 14987389

[16] Bonney KM, Engman DM (2008) Chagas heart disease pathogenesis: one mechanism or many? Curr Mol Med 8: 510–518. 18781958 10.2174/156652408785748004 PMC2859714

[17] Girones N, Fresno M (2003) Etiology of Chagas disease myocarditis: autoimmunity, parasite persistence, or both? Trends in Parasitology 19: 19–22. 12488221 10.1016/s1471-4922(02)00006-5

[18] Gutierrez FR, Guedes PM, Gazzinelli RT, Silva JS (2009) The role of parasite persistence in pathogenesis of Chagas heart disease. Parasite Immunol 31: 673–685. 19825107 10.1111/j.1365-3024.2009.01108.x

[19] Marin-Neto JA, Cunha-Neto E, Maciel BC, Simoes MV (2007) Pathogenesis of chronic Chagas heart disease. Circulation 115: 1109–1123. 17339569 10.1161/CIRCULATIONAHA.106.624296

[20] Rodriques Coura J, de Castro SL (2002) A critical review on Chagas disease chemotherapy. Mem Inst Oswaldo Cruz 97: 3–24. 11992141 10.1590/s0074-02762002000100001

[21] Gorla NB, Ledesma OS, Barbieri GP, Larripa IB (1989) Thirteenfold increase of chromosomal aberrations non-randomly distributed in chagasic children treated with nifurtimox. Mutation Research 224: 263–267. 2507913 10.1016/0165-1218(89)90165-1

[22] Gorla NB, Ledesma OS, Barbieri GP, Larripa IB (1988) Assessment of cytogenetic damage in chagasic children treated with benznidazole. Mutation Research 206: 217–220. 3140001 10.1016/0165-1218(88)90163-2

[23] Urbina JA (2010) Specific chemotherapy of Chagas disease: relevance, current limitations and new approaches. Acta Trop 115: 55–68. 19900395 10.1016/j.actatropica.2009.10.023

[24] Cancado JR (2002) Long term evaluation of etiological treatment of chagas disease with benznidazole. Rev Inst Med Trop Sao Paulo 44: 29–37. 11896410

[25] Pinto Dias JC (2006) The treatment of Chagas disease (South American trypanosomiasis). Ann Intern Med 144: 772–774. 16702594 10.7326/0003-4819-144-10-200605160-00012

[26] Zingales B, Andrade SG, Briones MR, Campbell DA, Chiari E, et al (2009) A new consensus for Trypanosoma cruzi intraspecific nomenclature: second revision meeting recommends TcI to TcVI. Mem Inst Oswaldo Cruz 104: 1051–1054. 20027478 10.1590/s0074-02762009000700021

[27] Villarreal D, Nirde P, Hide M, Barnabe C, Tibayrenc M (2005) Differential gene expression in benznidazole-resistant Trypanosoma cruzi parasites. Antimicrob Agents Chemother 49: 2701–2709. 15980339 10.1128/AAC.49.7.2701-2709.2005 PMC1168707

[28] Villarreal D, Barnabe C, Sereno D, Tibayrenc M (2004) Lack of correlation between in vitro susceptibility to Benznidazole and phylogenetic diversity of Trypanosoma cruzi, the agent of Chagas disease. Exp Parasitol 108: 24–31. 15491545 10.1016/j.exppara.2004.07.001

[29] Filardi LS, Brener Z (1987) Susceptibility and natural resistance of Trypanosoma cruzi strains to drugs used clinically in Chagas disease. Trans R Soc Trop Med Hyg 81: 755–759. 3130683 10.1016/0035-9203(87)90020-4

[30] Andrade SG, Magalhaes JB, Pontes AL (1985) Evaluation of chemotherapy with benznidazole and nifurtimox in mice infected with Trypanosoma cruzi strains of different types. Bull World Health Organ 63: 721–726. 3936634 PMC2536372

[31] Dias JC (2009) Elimination of Chagas disease transmission: perspectives. Mem Inst Oswaldo Cruz 104 Suppl 1: 41–45. 19753456 10.1590/s0074-02762009000900007

[32] Dias JC, Silveira AC, Schofield CJ (2002) The impact of Chagas disease control in Latin America: a review. Mem Inst Oswaldo Cruz 97: 603–612. 12219120 10.1590/s0074-02762002000500002

[33] Perez-Zuniga FJ, Seco EM, Cuesta T, Degenhardt F, Rohr J, et al (2004) CE-108, a new macrolide tetraene antibiotic. J Antibiot (Tokyo) 57: 197–204. 15152805 10.7164/antibiotics.57.197

[34] Rolon M, Seco EM, Vega C, Nogal JJ, Escario JA, et al (2006) Selective activity of polyene macrolides produced by genetically modified Streptomyces on Trypanosoma cruzi. Int J Antimicrob Agents 28: 104–109. 16844353 10.1016/j.ijantimicag.2006.02.025

[35] Miranzo D, Seco EM, Cuesta T, Malpartida F (2010) Isolation and characterization of pcsB, the gene for a polyene carboxamide synthase that tailors pimaricin into AB-400. Appl Microbiol Biotechnol 85: 1809–1819. 19707754 10.1007/s00253-009-2195-1

[36] Seco EM, Cuesta T, Fotso S, Laatsch H, Malpartida F (2005) Two polyene amides produced by genetically modified Streptomyces diastaticus var. 108. Chem Biol 12: 535–543. 15911374 10.1016/j.chembiol.2005.02.015

[37] Cardin GB, Mantha M, Jumarie C (2009) Resistance to cadmium as a function of Caco-2 cell differentiation: role of reactive oxygen species in cadmium- but not zinc-induced adaptation mechanisms. Biometals 22: 753–769. 19294337 10.1007/s10534-009-9223-6

[38] Amils R, Ramirez L, Sanz JL, Marin I, Pisabarro AG, et al (1989) The use of functional analysis of the ribosome as a tool to determine archaebacterial phylogeny. Can J Microbiol 35: 141–147. 2470480 10.1139/m89-021

[39] Barnabe C, Brisse S, Tibayrenc M (2000) Population structure and genetic typing of Trypanosoma cruzi, the agent of Chagas disease: a multilocus enzyme electrophoresis approach. Parasitology 120 (Pt 5): 513–526. 10.1017/s0031182099005661 10840981

[40] Telleria J, Biron DG, Brizard JP, Demettre E, Seveno M, et al (2010) Phylogenetic character mapping of proteomic diversity shows high correlation with subspecific phylogenetic diversity in Trypanosoma cruzi. Proc Natl Acad Sci U S A. 10.1073/pnas.1015496107 PMC2996636 21059959

[41] Mantel N (1967) The detection of disease clustering and a generalized regression approach. Cancer Res 27: 209–220. 6018555

[42] Telleria J, Barnabe C, Hide M, Banuls AL, Tibayrenc M (2004) Predominant clonal evolution leads to a close parity between gene expression profiles and subspecific phylogeny in Trypanosoma cruzi. Mol Biochem Parasitol 137: 133–141. 15279959 10.1016/j.molbiopara.2004.05.006

[43] Tibayrenc M, Ward P, Moya A, Ayala FJ (1986) Natural populations of Trypanosoma cruzi, the agent of Chagas disease, have a complex multiclonal structure. Proceedings Of The National Academy Of Sciences Of The United States Of America 83: 115–119. 3510428 10.1073/pnas.83.1.115 PMC322802

[44] Brisse S, Barnabe C, Tibayrenc M (2000) Identification of six Trypanosoma cruzi phylogenetic lineages by random amplified polymorphic DNA and multilocus enzyme electrophoresis. Int J Parasitol 30: 35–44. 10675742 10.1016/s0020-7519(99)00168-x

[45] Brisse S, Dujardin JC, Tibayrenc M (2000) Identification of six Trypanosoma cruzi lineages by sequence-characterised amplified region markers. Mol Biochem Parasitol 111: 95–105. 11087920 10.1016/s0166-6851(00)00302-9

[46] Soeiro MN, de Castro SL (2009) Trypanosoma cruzi targets for new chemotherapeutic approaches. Expert Opin Ther Targets 13: 105–121. 19063710 10.1517/14728220802623881

[47] Menna-Barreto RF, Salomao K, Dantas AP, Santa-Rita RM, Soares MJ, et al (2009) Different cell death pathways induced by drugs in Trypanosoma cruzi: an ultrastructural study. Micron 40: 157–168. 18849169 10.1016/j.micron.2008.08.003

[48] Nguewa PA, Fuertes MA, Valladares B, Alonso C, Perez JM (2004) Programmed cell death in trypanosomatids: a way to maximize their biological fitness? Trends in Parasitology 20: 375–380. 15246321 10.1016/j.pt.2004.05.006

[49] Debrabant A, Lee N, Bertholet S, Duncan R, Nakhasi HL (2003) Programmed cell death in trypanosomatids and other unicellular organisms. Int J Parasitol 33: 257–267. 12670511 10.1016/s0020-7519(03)00008-0

[50] Lee N, Bertholet S, Debrabant A, Muller J, Duncan R, et al (2002) Programmed cell death in the unicellular protozoan parasite Leishmania. Cell Death Differ 9: 53–64. 11803374 10.1038/sj.cdd.4400952

[51] Liu W, Apagyi K, McLeavy L, Ersfeld K (2010) Expression and cellular localisation of calpain-like proteins in Trypanosoma brucei. Mol Biochem Parasitol 169: 20–26. 19766148 10.1016/j.molbiopara.2009.09.004

[52] Olego-Fernandez S, Vaughan S, Shaw MK, Gull K, Ginger ML (2009) Cell morphogenesis of Trypanosoma brucei requires the paralogous, differentially expressed calpain-related proteins CAP5.5 and CAP5.5V. Protist 160: 576–590. 19656721 10.1016/j.protis.2009.05.003

[53] Sangenito LS, Ennes-Vidal V, Marinho FA, Da Mota FF, Santos AL, et al (2009) Arrested growth of Trypanosoma cruzi by the calpain inhibitor MDL28170 and detection of calpain homologues in epimastigote forms. Parasitology 136: 433–441. 19250597 10.1017/S0031182009005629

[54] Giese V, Dallagiovanna B, Marchini FK, Pavoni DP, Krieger MA, et al (2008) Trypanosoma cruzi: a stage-specific calpain-like protein is induced after various kinds of stress. Mem Inst Oswaldo Cruz 103: 598–601. 18949332 10.1590/s0074-02762008000600015

[55] Lee N, Gannavaram S, Selvapandiyan A, Debrabant A (2007) Characterization of metacaspases with trypsin-like activity and their putative role in programmed cell death in the protozoan parasite Leishmania. Eukaryot Cell 6: 1745–1757. 17715367 10.1128/EC.00123-07 PMC2043384

[56] Kosec G, Alvarez VE, Aguero F, Sanchez D, Dolinar M, et al (2006) Metacaspases of Trypanosoma cruzi: possible candidates for programmed cell death mediators. Mol Biochem Parasitol 145: 18–28. 16213036 10.1016/j.molbiopara.2005.09.001

[57] Hacker G, Paschen SA (2007) Therapeutic targets in the mitochondrial apoptotic pathway. Expert Opin Ther Targets 11: 515–526. 17373881 10.1517/14728222.11.4.515

[58] Lewis MD, Ma J, Yeo M, Carrasco HJ, Llewellyn MS, et al (2009) Genotyping of Trypanosoma cruzi: systematic selection of assays allowing rapid and accurate discrimination of all known lineages. Am J Trop Med Hyg 81: 1041–1049. 19996435 10.4269/ajtmh.2009.09-0305 PMC2825677

[59] Sturm NR, Campbell DA (2010) Alternative lifestyles: the population structure of Trypanosoma cruzi. Acta Trop 115: 35–43. 19695212 10.1016/j.actatropica.2009.08.018

[60] Tibayrenc M, Ayala FJ (1988) Isozyme variability of Trypanosoma cruzi, the agent of Chagas’ disease: genetical, taxonomical and epidemiological significance. Evolution 42: 277–292. 28567853 10.1111/j.1558-5646.1988.tb04132.x

[61] Tibayrenc M, Ayala FJ (2002) The clonal theory of parasitic protozoa: 12 years on. Trends in Parasitology 18: 405–410. 12377258 10.1016/s1471-4922(02)02357-7

[62] Garcia ES, Azambuja P (1991) Development and interactions of Trypanosoma cruzi within the insect vector. Parasitol Today 7: 240–244. 15463507 10.1016/0169-4758(91)90237-i

[63] Risso MG, Garbarino GB, Mocetti E, Campetella O, Gonzalez Cappa SM, et al (2004) Differential expression of a virulence factor, the trans-sialidase, by the main Trypanosoma cruzi phylogenetic lineages. J Infect Dis 189: 2250–2259. 15181573 10.1086/420831

[64] Ruiz RC, Favoreto S Jr, Dorta ML, Oshiro ME, Ferreira AT, et al (1998) Infectivity of Trypanosoma cruzi strains is associated with differential expression of surface glycoproteins with differential Ca2+ signalling activity. Biochem J 330 (Pt 1): 505–511. 10.1042/bj3300505 PMC1219166 9461549

[65] Piazza RM, Borges MM, Kloetzel JK, Stolf AM (1996) Reactivity of Trypanosoma cruzi strains with peanut agglutinin (PNA) correlates with number of in vitro infected host cells. Acta Trop 61: 41–50. 9133163 10.1016/0001-706x(95)00139-6

[66] Croft SL, Snowdon D, Yardley V (1996) The activities of four anticancer alkyllysophospholipids against Leishmania donovani, Trypanosoma cruzi and Trypanosoma brucei. J Antimicrob Chemother 38: 1041–1047. 9023651 10.1093/jac/38.6.1041

[67] Luna KP, Hernandez IP, Rueda CM, Zorro MM, Croft SL, et al (2009) In vitro susceptibility of Trypanosoma cruzi strains from Santander, Colombia, to hexadecylphosphocholine (miltefosine), nifurtimox and benznidazole. Biomedica 29: 448–455. 20436996

[68] Toledo MJ, Bahia MT, Carneiro CM, Martins-Filho OA, Tibayrenc M, et al (2003) Chemotherapy with benznidazole and itraconazole for mice infected with different Trypanosoma cruzi clonal genotypes. Antimicrob Agents Chemother 47: 223–230. 12499195 10.1128/AAC.47.1.223-230.2003 PMC149031

